# Multipurpose RNA maturation factors dysregulate multiple mRNA processing steps simultaneously and provide new therapeutic opportunities

**DOI:** 10.1080/15476286.2025.2503040

**Published:** 2025-06-09

**Authors:** Sunirmal Paira, Katherine L. B. Borden

**Affiliations:** a Department of Pharmacology, Northwestern University, Chicago, IL, USA; b Robert H Lurie Comprehensive Cancer Center, Feinberg School of Medicine, Department of Pharmacology, Northwestern University, Chicago, IL, USA

**Keywords:** Translation, export, splicing, eIF4E, SF3B1, multi-tasking

## Abstract

mRNAs undergo a series of chemical modifications to become competent for nuclear export and translation. This is referred to as mRNA maturation or processing and includes capping, splicing, and 3’end formation. These steps can be hijacked in cancer to alter proteins’ forms and levels in the absence of mutation or changes to transcript levels. Here, we focus on an emerging idea that some factors act in multiple processing events and that their dysregulation in both their canonical and noncanonical functions contributes to cancer with a focus on Acute Myeloid Leukaemia (AML). As examples, we discuss the eukaryotic translation initiation factor (eIF4E), splice factor 3 complex B subunit 1 (SF3B1), U2 small nuclear auxiliary factor (U2AF1), and associated factors. These physically interact with each other and play roles in splicing, export, and translation. Malignant dysregulation of this mRNA processing-export-translation axis diversifies the proteome to support cancer. Finally, we discuss the simultaneous dysregulation of mRNA processing in malignancy and related therapeutic development.

## Overview of mRNA processing and its relevance to cancer

**Figure 1.**
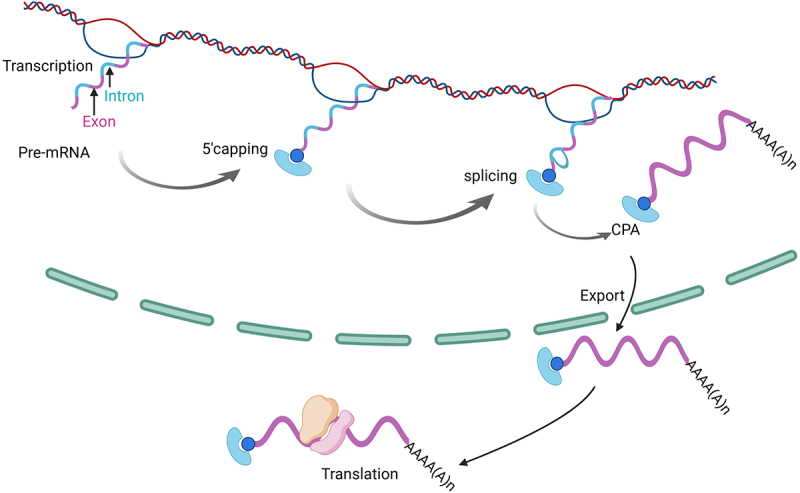
Schematic model depicting major stages of mRNA processing. Note that major processing steps are coordinated with transcription (top threads indicate DNA). Canonically, mRNA processing starts with capping, then splicing, cleavage and polyadenylation (CPA), followed by mRNA export to the cytosol and translation on the ribosomes. Intronic mRNA sequences are indicated in blue and exonic sequences in pink. The cap-binding factors CBC or eIF4E is shown in blue. Given that both can bind nuclear mRNAs, they are not distinguished here. The blue ball is cap. For simplicity, all relevant factors are not shown. Figure was generated in Biorender.

All cellular activities are ultimately reliant on the production of relevant proteins. The generation of a cellular proteome attuned to the necessary cellular context depends on the cell’s capacity to produce mature translation-competent mRNAs that have accurately undergone co-/post-transcriptional processing events [1–4] (Figure 1). mRNA processing is a series of chemical modifications to mRNAs that produce mature transcripts ready for different downstream processes, including translation, degradation, storage, etc. Modifying these steps in response to environmental or intracellular cues can alter transcript diversity, thereby impacting the final cellular proteome [1–4]. In cancer cells, mRNA processing can be hijacked to produce a malignant proteome without any detectable alteration to gene expression levels [1].

The major steps in mRNA processing include the addition of the methyl-7-guanosine (m^7^G) cap on its 5’end during a process known as capping, excision of introns from pre-mRNA during splicing, 3’end formation referred to us as cleavage and polyadenylation (CPA), all of which ultimately produce mature, functional mRNA transcripts [1–4] (Figure 1). mRNA processing is often closely coordinated with transcription, suggesting that a physical organization is relevant for the flow of mRNAs between the different processing steps [2,3]. However, some processing steps, such as splicing and CPA, can also occur distal to sites of transcription [5,6]. An imbalance in the regulation of the RNA processing steps or lack of coordination with nuclear mRNA export or translation could substantially impact functional diversity in the proteome [1–4,7–10] Dysregulation of specific events can have widescale impacts, such as alternative splicing, which generates different RNA isoforms from a single gene, thereby generating proteins with various functions from the same transcriptome or reducing protein levels by impacting mRNA stability by introducing premature stop codon and leading to mRNA degradation [7,8]. The reprogramming of splicing patterns is of significant biomedical relevance due to the role of splicing in the incidence of several diseases, including cancer, which can be characterized by splice factor (SF) mutations and/or SF dysregulation [7–11]. A common observation is that these factors only influence a subset of RNAs despite their general role in splicing.

Dysregulation of capping, splicing, export, and translation are all observed and can contribute to many cancers [7–16]. In this review, we discuss factors that play roles in normal and aberrant mRNA processing in cancer, focusing on acute myeloid leukaemia (AML) and related haematological malignancies. This is a broad area, and thus, we focus on exemplar factors that have the capacity to act in multiple steps in the mRNA processing-export-translation axis, including eIF4E, SF3B1 and U2AF1. All of these factors are dysregulated in cancer [8–10,16–18]. We discuss how these factors interact physically, thereby revealing an intricate interplay between related mRNA processing events, export, and translation. We also discuss their canonical and non-canonical functions, which can influence the production of oncogenic proteins, increasing motility, proliferation, and apoptotic survival, which are key drivers of malignancy. Finally, we discuss how the simultaneous dysregulation of mRNA processing contributes to malignancy and how understanding the associated molecular mechanisms can be leveraged to therapeutic advantage.

## eIF4E: a cap-binding protein implicated in many steps of mRNA processing

eIF4E is dysregulated in a wide array of cancers, including AML [15,18–26]. In mouse models, eIF4E overexpression can drive malignancy [27–30]. eIF4E drives cell motility, rescue from apoptotic stimuli and loss of contact inhibition in immortalized cell lines [27,29,31–36]. eIF4E elevation is associated with metastatic disease and poor outcomes in patients [20,23–25,37–40]. High elevation and nuclear enrichment of eIF4E are associated with poor outcomes in AML patients [18,19,40,41]. Multiple early phase clinical trials have shown the capacity to target eIF4E correlates with objective clinical responses, including complete remissions in some patients [18,19,24,41]. We will discuss the therapeutic targeting of eIF4E in later sections. Here, we discuss molecular activities of eIF4E that underpin its oncogenic potential.

**Figure 2.**
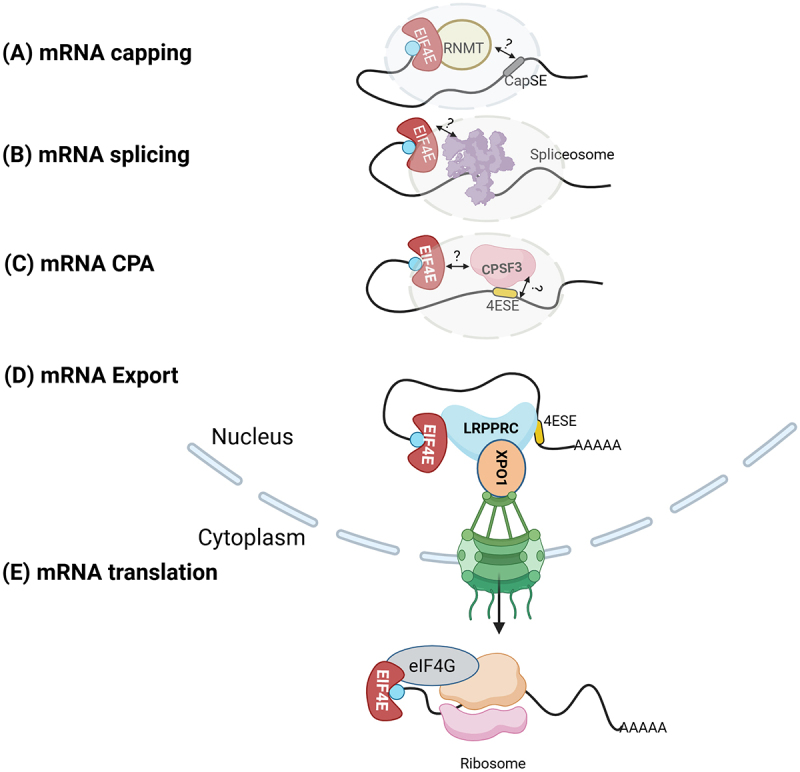
eIF4E acts as a master regulator by influencing mRNA Capping (a), Splicing (b), Cleavage and polyadenylation (CPA) (c), export (d), and cytoplasmic translation (e) [4,34,40,43,44,63,72–74]. The green barrel represents the nuclear pore complex, blue bars are the nuclear membrane. In all the major RNA processing events, eIF4E interacts with mRNA processing machinery factors. eIF4E influences the production of some of these factors by regulating the export and translation of their mRNAs. Gray circles and question marks denote that it is unknown whether the factors directly interact or are mediated by unknown cofactors. The blue ball is the cap. For simplicity, all relevant factors are not shown. See the text for details. Figure was generated in Biorender.

eIF4E is a highly conserved protein found in both the nucleus and cytoplasm of a wide variety of eukaryotic organisms, ranging from yeast to humans, with well-defined roles in translation and nuclear mRNA export of specific subsets of transcripts [15,17,30,34,42–48]. The binding of eIF4E to the methyl-7-guanosine cap found on the 5’end of mRNAs is crucial for its activity in both the nucleus and cytoplasm [47,49]. In the canonical translation initiation model, eIF4E binds capped mRNAs using its cap-binding pocket and the platform protein eIF4G through its dorsal surface [42]; the association of eIF4G with eIF3 promotes the assembly of 43S preinitiation complex (PIC) and eIF4E, including eIF4E-bound capped mRNA [50] (Figure 2). Following assembly, the 43S PIC scans for a start codon, and then 60S ribosome subunit engages to form the 80S ribosome subunit, thereby initiating translation [47,51,52]. For selected mRNA, overexpression of eIF4E increases the translation efficiency (more ribosomes per transcript), which in turn causes an elevation of corresponding oncogenic protein levels without any transcriptional changes [47,53]. However, the overexpression of eIF4E does not increase the translation efficiency of all capped mRNAs [47]. The specificity of selected mRNA transcripts is yet to be well dissected but is usually characterized by highly structured GC-rich elements within the 5’UTR [47,54]. Other elements that sensitize mRNAs to eIF4E are the **c**ytosine-**e**nriched **r**egulator of **t**ranslation (CERT) [55] and the **p**yrimidine-**r**ich **t**ranslational **e**lements (PRTE) [56]. Elements such as these USER codes (**u**ntranslated **s**equence **e**lements for **r**egulation) [57–59] provide selectivity to oncogenic mRNAs, e.g. *VEGF, Cyclin D1, ODC, c-Myc* that code for proliferation and oncogenic properties [47]. In this way, mRNAs encoding proteins participating in the same biological activities contain the same cis-acting elements known as USER codes in what is known as the RNA regulon [57–59]. These engage specific RNA-binding proteins (RBPs) or miRNAs, which allows co-regulation of the mRNAs containing the same USER code. Transcripts usually contain multiple USER codes enabling collaborative or competitive RBP interaction. USER codes are generally specific for different processing events, including sequestration or stability and, here, translation [55–59].

eIF4E is also found in the nucleus [15,34,60–62]. Its best-described nuclear role is in the nuclear export of target mRNAs containing the eIF4E sensitivity elements (4ESE) USER code in their 3’UTR [43–45]. The 4ESE-containing mRNAs are bound by eIF4E through their m^7^G cap, and LRPPRC (**L**eucine-**r**ich **p**entatrico**p**eptide **r**epeat **C**-terminus) directly binds the 4ESE RNA and eIF4E simultaneously [45,63] (Figure 2). LRPPRC also directly interacts with nuclear export receptor CRM1/XPO1, forming an eIF4E-Cap-4ESE-RNA-LRPPRC-XPO1 complex [45,63]. Thus, LRPPRC acts as an RNA export assembly platform and promotes export by bridging the eIF4E-4ESE RNA and XPO1 interaction as seen by NMR, size exclusion chromatography, and biochemical assays [63]. XPO1 ferries the complex to transit through the nuclear pore complex (NPC) [63] (Figure 2). Export of eIF4E-sensitive mRNAs, e.g. *Cyclin D1, Pim-1 and c-MYC*, but not bulk mRNA [33,43,44] are repressed by the loss of LRPPRC [45,64]. Studies using mutants of eIF4E showed that export activities are important for its oncogenic potential [33,34,43,44,65,66]. The S53A mutant of eIF4E is impaired in mRNA export but active in translation with no reduction of cap-binding capacity or changes to its structure as seen by NMR [27,34,65,67–69]. However, the S53A mutant does not modulate contact inhibition or rescue cells from apoptotic stimuli [27,34]. By contrast, the W73A mutant is active in mRNA export and does not influence translation due to impaired eIF4G binding [17,33,34,47,66,70]. The W73A mutant acted like wild-type eIF4E in terms of reduced contact inhibition and rescued cells from apoptotic stimuli consistent with having oncogenic potential [17,31–34,44,65]. Thus, the capacity of eIF4E to promote RNA export substantially contributes to its oncogenic potential.

Another nuclear activity of eIF4E involves alternative splicing. eIF4E dysregulation modulates altered splicing for ~4000 mRNAs in high-eIF4E AML patients compared to normal eIF4E patients and nearly 1000 in eIF4E overexpressing cell lines relative to vector control [40]. The overexpression of eIF4E alone is sufficient to modulate splicing events without the induction of any known SF mutations [40]. Depending on the specific event and transcript, eIF4E can promote or repress exon inclusion. eIF4E mainly influences the exon skipping (ES) and inclusion of mutually exclusive exons (MXE). eIF4E interacts with splice factors, e.g. PRPF6, PRPF8, PRPF19, PRPF31, SF3B1, U2AF1, U2AF2, SNRNP200 and their corresponding snRNAs (small nuclear RNA) in an RNA and cap-dependent manner in normal U2OS cells as well as in high-eIF4E AML cells, suggesting eIF4E uses its cap-binding activity to bind target mRNAs [40]. eIF4E also increased the splice factors’ protein production by promoting the nuclear export and translation in some SF-encoding mRNAs without affecting their transcription or mRNA stability [40]. eIF4E did not affect snRNA levels and thus likely affects the composition of spliceosomes rather than their number. Nuclear eIF4E interacted with both the intron-containing pre-mRNAs and their mature forms for certain transcripts, which suggests that eIF4E has a direct role in chaperoning mRNAs through splicing [40]. A comparison of nuclear eIF4E RNA immunoprecipitation (RIP)-seq data to the RNAs identified as eIF4E-dependent splicing targets in high-eIF4E AML patients revealed that ~1,300 transcripts were shared between these two groups, suggesting that all transcripts are not direct targets of eIF4E-dependent alternative splice targets. Moreover, for those interactors that are not splicing targets, they are presumably targets of other eIF4E nuclear processing activity such as export, capping or polyadenylation [40]. Overall, these findings suggest a two-tier system of splicing regulation: (i) Some eIF4E-dependent splicing changes result from eIF4E modulating SF production by promoting export and translation efficiency of RNAs encoding SFs, and (ii) eIF4E physically interacts with splicing factors. In the previous work, eIF4E was implicated in the splicing of *SXL* with a physical interaction with the U2 snRNP in *Drosophila*, suggesting that the connection between eIF4E and splicing is evolutionarily conserved [71].

In addition to the above-mentioned role, eIF4E is also implicated in mRNA capping and CPA [72,73]. eIF4E controls the production of the machinery at the mRNA export and sometimes translation level. Additionally, eIF4E physically interacts with the capping enzyme RNMT and the CPSF3 and CPSF1 constituents of the CPA machinery [72–74]. The USER code for capping differs from the 4E-SE and is known as the Cap Sensitivity Element CapSE. The CapSE recruits RNMT to target transcripts [73]. Thus, eIF4E is positioned to couple nuclear mRNA processing and RNA export with cytoplasmic translation (Figure 2).

Interestingly, eIF4E influences the activity of some SFs by regulating their mRNA export and protein production, as mentioned above. Some of these SFs, like SF3B1 and U2AF1, also physically interact with eIF4E in the nucleus [40]. Like eIF4E, these SFs also play diverse roles in mRNA processing events beyond splicing [75–77]. It appears very likely that these multipurpose factors produce a complex network by coordinating different mRNA processing activities simultaneously with the aid of the master modulator eIF4E. Consistent with its role as a multi-tasking protein, eIF4E has been found to associate directly or indirectly with >80 proteins, including components of the nuclear export machinery, spliceosome, splicing-related factors, and CPA factors, thereby influencing major events of mRNA processing simultaneously [4].

## Canonical functions of splicing factors associated with AML

**Figure 3.**
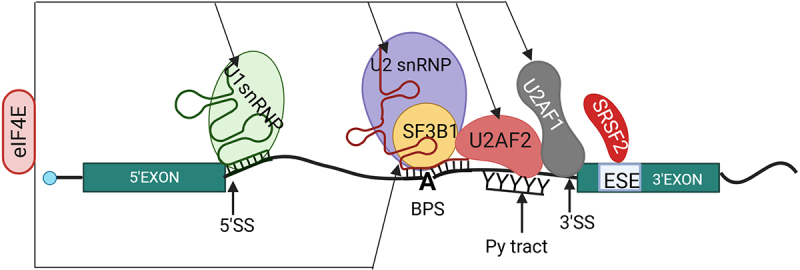
The schematic model depicts the splice factors that regulate alternative splicing. Introns are narrow black lines, and exons green rectangles. The green stem loop structures represent U1 snRNA, and the red stem loop structures U2 snRNA. At the 5’Splice site (5’SS), U1 snRNP binds with pre-mRNA, SF3B1 and U2snRNP binds at the BPS (branch point sequence), and U2AF1 binds at the 3’Splice site(3’SS); U2AF2 binds at the Poly pyrimidine tract (Py-tract). Note that the binding of splicing factors at the proper sequence of pre-mRNA is essential for regulating accurate splicing. SRSF2 binds ESE (Exon Splicing Enhancer) present usually in exons and modulates the splicing. As shown in the figure, the black arrows indicate eIF4E is found to be associated with SF3B1, U2AF1, U2AF2, U1, and U2 snRNP [40] by immunoprecipitation, whether the interaction is direct or indirect is unknown. Figure was generated in Biorender.

During mRNA splicing, non-coding introns are removed from the transcripts, and the exons must be ligated together [78,79]. Splicing is primarily executed by the major spliceosome, consisting of more than 150 proteins and five small uridine-rich nuclear RNAs (U1, U2, U4, U5, and U6), each of which is associated with a large number of proteins forming small nuclear ribonuclear particles (snRNPs) [78,79]. Different snRNPs and their associated snRNAs bind within the intronic sequences through multiple steps to conduct the splicing. Introns contain short consensus sequences at the 5' splice site, while the 3' splice site includes three key elements: the branchpoint sequence (BPS), a polypyrimidine tract (Py-tract), and an AG dinucleotide at the junction between the intron and exon, which together guide splicing [78,79]. The spliceosome assembly and splicing start with the binding of U1 at the 5’ splice site, binding of SF1 at the BPS and U2AF complex (including U2AF1 and U2AF2) at the Py-tract and 3’ splice site, respectively. Then, SF1 is displaced by the binding of U2snRNP at the BPS. The SF3b1 component of U2snRNP recognizes BPS and reinforces the base pairing between U2snRNA and the BPS of pre-mRNA (Figure 3). These initial steps of spliceosome assembly and recognition of splice sites are important for determining the boundaries of transcripts that will be spliced out, producing the mature mRNAs. Alternative splicing can arise from an alteration in spliceosome assembly or mutation in splicing components or altered expression of the non-spliceosomal RNA binding proteins (RBP), which are responsible for recognizing the cis-acting elements [80]. These cis-acting elements, which modulate splicing and are located in the exons, are known as an exonic splicing enhancer (ESE) or repress splicing and are located within the exon that is known as an exonic splicing silencer (ESS). RBPs like SRSF2 bind to ESEs to regulate splicing by promoting the binding of U1 and U2snRNPs at the 5’ss and 3’ss [80]. Almost all multiple exonic sequences (∼95%) undergo alternative splicing (AS), where a particular 3’ss can be joined to different 5’ss or vice versa [81,82]. The main AS events are the skipped exons, retained introns, alternative 3’ and 5’ splice sites, or mutually exclusive exons [80,83].

Here, we focus on SF3B1, U2AF1, and SRSF2 splice factors as they are mutated in a subset of AML patients [16,84,85], are regulated by and interact with eIF4E [40], and play roles beyond splicing [75–77]. These SF mutations are mostly associated with myelodysplastic syndromes (MDS) but can, in some cases, drive progression to AML [7,16,86,87]. In MDS and AML patients, the SF mutations are associated with some other epigenetic mutations, like SF3B1 is associated with DNMT3A mutations, SRSF2 with RUNX1, IDH1/2 and ASLX1 mutations, and U2AF1 with ASXL1 and DNMT31[16]. Some fusion genes are also found due to chromosomal aberrations in paediatric and adult AML cases. The most frequent fusion genes in AML are *RUNX1::RUNX1T1, PML::RARA, ZNF292::PNRC1, NUP98::NSD1, CBFB::MYH11, KMT2A::MLLT4, KMT2A::MLT3, KMT2A::MLLT10* and *DEK::NUP214 [84]*. These fusion gene mutations are rarely associated with altered splicing in AML.

While less than 10% of AML patients have splice factor mutation, 30% of AML patients exhibit AS profiles, suggesting that there are alternative means to drive dysregulated splicing [85]. In some solid tumours, the levels of SRSF1 and SRSF6 are upregulated, though the reason for this upregulation of splicing factors is context-specific [7]. For example, the transcription of SRSF1 is directly activated by MYC [88]. MYC is also highly overexpressed and dysregulated in AML. High MYC expression is associated with a shorter MDS progression to AML [88]. Thus, cooperation between MYC and SRSF1 may lead to wide alternative splicing changes in AML [89,90]. Additionally, in some secondary AML specimens, SF3B1 and a component of another splicing complex, PRPF6, are elevated compared to healthy volunteers [91]. In these malignancies, the underlying mechanism of this elevation is unknown. As we described above, eIF4E also upregulates protein levels of factors such as SF3B1 and PRPF proteins, but it is unknown how widespread its contribution to alternative splicing and production of these factors is in AML.

**Figure 4.**
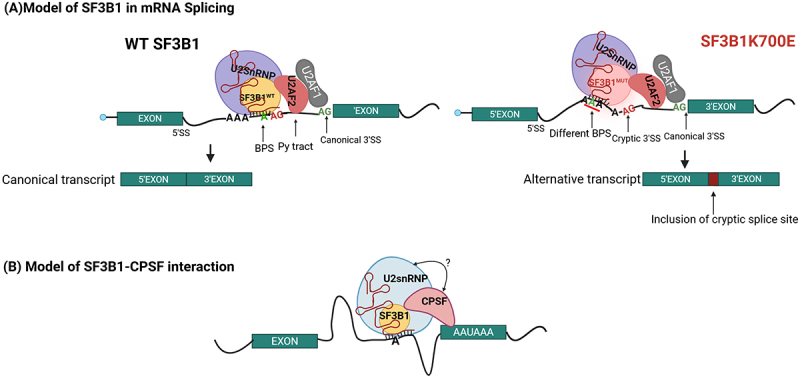
Schematic model depicting the canonical and moonlighting roles of SF3B1. (a) During canonical splicing, Wild type (WT) SF3B1 recognizes BPS while mutant SF3B1(K700E) recognizes a different sequence as a branch point, enabling usage of cryptic 3’SS, leading to alternate transcripts. (b) Beyond splicing, SF3B1 was found to associate with the CPSF complex for 3’end processing of some mRNAs [108]. AAUAAA represents the Polyadenylation signal [150]. The question mark indicates whether direct or indirect interaction between U2snRNP and CPSF is unknown. Figure was generated in Biorender.

Of these three splicing factors, much interest has been focused upon SF3B1. SF3B1 directly interacts with the U2 snRNA and BPS on pre-mRNA and forms a stable complex with U2AF2, which directs the selection of the 3’ splice site during the pre-mRNA splicing process [92] (Figure 3). Commonly, SF3B1 mutations are found in a form of MDS known as refractory anaemia with ring sideroblasts and refractory cytopenia with multilineage dysplasia [16,93,94]. The predictive outcome of SF3B1 mutations varies based on the specific mutation and other clinical aspects. For example, the SF3B1 K700E mutation is linked to ring sideroblasts and associated with better prognosis [95]. This SF3B1 K700E mutant uses non-canonical 3’splice sites by recognizing non-canonical BPSs [96] (Figure 4). By contrast, the SF3B1 K666N mutation is associated with disease progression, and this mutation causes dysregulation due to the use of non-canonical 3’splice sites [96,97]. For example, SF3B1 K700E mutant reduces the RNA and protein level of *MAP3K7* due to usage of cryptic 3’ss upstream of canonical 3’ss, which ultimately leads to nonsense-mediated mRNA decay of *MAP3K7* [98]. Thus, the resulting decreased levels of *MAP3K7* transcripts are responsible for p38/MAPK deactivation and greater and faster downregulation of GATA1, ultimately leading to apoptotic erythroid cell death and anaemia in MDS patients with SF3B1 K700E mutation [98]. A comparison of splicing profiles arising in high-eIF4E cells relative to AML cells with SF3B1 mutation reveals that those from eIF4E overexpressing cells are distinct but partially overlap with those seen with SF3B1 mutations [40]. There were ~50 shared targets out of ~4000 eIF4E-dependent splicing targets from AML patient specimens compared with SF3B1 mutation [40]. However, a significant portion of SF3B1 targets (47 out of 83) also overlapped with eIF4E-dependent splicing targets from AML patients [40]. SF3B1 mutations primarily altered 3' splice site selection [99], while eIF4E overexpression mainly influenced exon skipping and mutually exclusive exon events [40].

**Figure 5.**
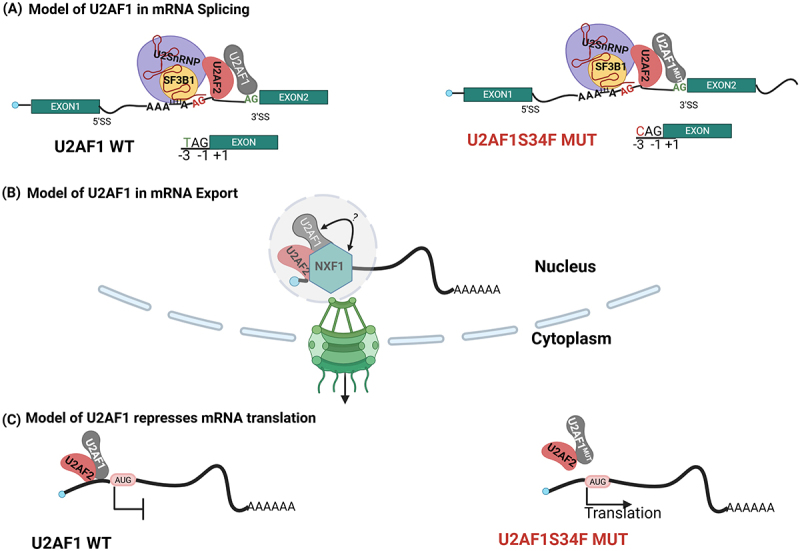
Schematic representation showing U2AF1 acts in different mRNA processing events and translational regulation. (a) During splicing, Wild type (WT) U2AF1 recognizes canonical 3’ss with a preference for the sequence TAG; by contrast, the S34F mutant preferentially binds alternative 3’SS with a favourable inclusion of exons with a C nucleotide at the -3 position, leading to alternative splicing [101]. (b) U2AF1 also interacts with NXF1, implicating it in NXF1-mediated mRNA export [77]. The gray circle and the question mark denote that it is unknown whether the factors directly interact or are mediated by unknown cofactors. The green barrel represented the nuclear pore complex, blue bars are the nuclear membrane. (c) U2AF1 is also localized in the cytosol and represses the translation of some mRNAs like IL8, while the S34F mutant does not bind the transcripts and thus derepresses the translation [76]. Figure was generated in Biorender.

U2AF1 is another major component of the spliceosome, which is associated with poor prognosis in both MDS and AML [100]. U2AF1 recognizes the AG dinucleotide at the 3’splice site of intronic pre-mRNA through its zinc finger domain and forms a heterodimer complex with U2AF2 through its U2AF homology motif (UHM) [101] (Figure 5). In MDS and AML patients, mutations of S34F and Q157R in U2AF1 are common, and both are located within their zinc finger domains, which leads to an alteration in the RNA binding affinity of U2AF1 and recognition AG dinucleotide [101]. These mutations alter splicing in a sequence-dependent manner by identifying nucleotides around the AG dinucleotides at the 3’ splice site [101]. U2AF1 S34F mutation tends to include exons bearing a C nucleotide at the −3 position of 3’SS (Figure 5). On the other hand, Q157R mutant promotes the inclusion of exons by recognizing G nucleotide at the +1 position instead of A [101].

Finally, SRSF2 mutations are found in a subset of MDS and AML patients. SRSF2 mutations are found in ~10% of MDS, ~30–50% of chronic myelomonocytic leukaemia (CMML), and ~2% of AML [102]. In general, SRSF2 acts as a splicing activator by binding to ESE in the exon sequence of target RNA [103]. SRSF2 binds to the ESE through its RNA recognition motif (RRM) and promotes the recruitment of U1 and U2 snRNPs to 5’ and 3’ splice sites (Figure 3) [104,105]. The most common mutation in SRSF2 is the P95H mutation, which is in the linker region between the RRM and arginine/serine (RS) rich domain [103–105]. The P95H mutation leads to altered binding preference of SRSF2 to RNA motif of ESE in the targeted RNA [105]. Wild-type SRSF2 efficiently binds at the CCNG or GGNG motif of mRNA within the ESE through a stacking interaction, while the P95H mutant preferentially forms strong hydrogen bonds with the CCNG motif of ESE. H-bonds are generally stronger than base stacking interaction, thereby enhancing the inclusion of the exons with C-rich (CCNG) ESEs, which in turn leads to altered splicing [105,106]. SRSF2 mutations are associated with poor prognosis in MDS and AML, making them a potential therapeutic target [105].

## Splicing factors can moonlight in other RNA processing steps and in translation

The SF3b complex and U2AF1 have roles in multiple stages of the RNA life cycle. For example, the SF3b complex acts in mRNA export through interactions with the bulk mRNA export complex, including bulk export factors TREX and NXF1, potentially coupling splicing and export [107]. This function is independent of U2 snRNP association by SF3B1 [107]. SF3B1 knockdown inhibits the export of bulk mRNA as measured by oligo-dT *in situ* hybridization. SF3B1 promotes mRNA export by recruitment of THO/TREX complex [107]. Microinjection of three plasmids (*HSPA1A, FOXC2* and *CLDN3*) and FISH studies found that SF3b knockdown affected the export of these mRNAs [107]. eCLIP data revealed that SM (SF3b binding motif) can also be found in one-third of naturally intronless mRNA and two-thirds of intron-containing mRNAs. This motif promotes the binding of SF3B1, which enhances the export of intronless (*H1-H2AG, H1-H3H, HSPA1A, RHOB*) and mature (*UAP56, FOS, FOXF2*) transcripts through THO/TREX recruitment [107]. Although eIF4E does not use the TREX/NXF1 export pathway [63], eIF4E elevation of SF3B1 may also influence the SF3B1-dependent RNA export indirectly. The interaction of the SF3b subunits with the components of the CPA machinery is another example where SF3b acted in multiple mRNA processing events [108] (Figure 4). Other components of SF3b interact with the translation machinery in the cytoplasm [109]. SF3b4 binds to p180, a factor involved in ER translation and acts in the translation of COL1A1 in a manner dependent on its 5’ untranslated region [109]. This 5’UTR is required for ER localization of SF3b4 and p180-dependent ER translation, bridging them together [109]. The abundant expression of ER-localized SF3B4 and p180 cooperatively promotes the targeting of specific mRNA to the ER and their increased translation [109]. SF3b components were also found to be associated with the proteasome [75]. SF3b3 interacts with the Cullin RING Ubiquitin ligase complex (CRL) and negatively regulates the assembly and ligase activity of CRL [110]. SF3b3 binds to different Cullin proteins and competes with Skp1-F-box protein for Cullin1 binding and negatively affects ligase-mediated substrate degradation [110]. The relevance of its RNA-binding activity to this novel function is unknown. The physical association of SF3B1 and eIF4E in the nucleus [40] might be paralleled in the cytoplasm; this warrants further study.

Similar to SF3B1 and eIF4E, U2AF1 is found in both the nucleus and the cytoplasm. In the cytoplasm, it binds mature mRNA and is involved in translation inhibition and mRNA export [76,77] (Figure 5). The U2AF1/2 heterodimer directly binds proximal to the start codon of some mRNAs, thereby repressing their translation. Interestingly, the S34F mutation in U2AF1 is associated with MDS only, leading to minor dysregulation of its splicing activity [76]. However, this mutation appears to impair its translation inhibitory activities substantially in refractory and relapsed AML [76]. This is not surprising as this mutation is found in its nucleic-acid binding domain, which it uses in both 3’ splice site recognition and translation activities [76]. For example, the S34F mutation is associated with a loss of translation repression of *IL8* mRNA by U2AF1, with no effects of *IL8* splicing, leading to elevated IL8 protein levels [76] (Figure 5). Additionally, U2AF1 binds to TAP/NXF1 and facilitates the association of TAP/NXF1 with export-targeted RNA [77]. This appears to occur at splice sites consistent with the traditional view that RNA export is coupled via proximity to transcription sites [77]. In this way, splice factors can act as a multi-tasking protein and influence mRNA processing, export, and translation, thereby connecting nuclear events and cytosolic translation by acting at different locations within the cell. Thus, these splice factors can make a circuit with their multipurpose RNA processing ability, simultaneously affecting different mRNA processing events and generating a more diversified proteome.

## Therapeutic targeting of malignant RNA processing

Disrupted RNA processing plays a key role in developing AML and other cancers, making them major therapeutic targets. SF3B1 and the SF3b complex have been specifically in focus for developing splicing modulators. Key compounds in this class include Spliceostatin A (SSA), sudemycin, pladienolide B (PB), E7107, H3B-8800, and herboxidiene analogues, all of which directly affect SF3b complex [111] (Table 1). Importantly, genetic reduction of SF3B1 does not impact all splicing events nor does pharmacological targeting, but rather reprogrammes the splicing of specific events [112]. Among all the SF3B targeting compounds, only E7107 and H3B-8800 have gone into clinical trials [113,114]. E7107 binds the SF3b complex and blocks spliceosome assembly by preventing the binding of U2snRNP to the pre-mRNA [115] and was the first therapy used to target splicing in patients by inhibiting the activity of spliceosome [113,116]. This first clinical trial was in solid tumours, and the best response was stable disease, but the trial was put on hold due to vision loss in two patients [113,116]. Following this, H3B-8800 was evaluated in a Phase I clinical trial for 84 AML, MDS, and CMML patients, where there was no selection of patients based on their SF3B1 mutation status [114] (Table 1). While no objective clinical responses were observed, 15% of patients had reduced transfusion dependency and acceptable toxicity. Molecular analyses confirmed that H3B-8800 effectively modulated the splicing of some example pre-mRNA in some of these patients [114]. Further studies are needed to understand the limited clinical benefits of these therapies, i.e. does this arise because only targeting of the splicing of select mRNAs and/or are these compensatory mechanisms allowing cells to override this therapeutic strategy is observed.

**Table 1.** Summary of inhibitors affecting factors involved in post-transcriptional regulation of mRNAs.

Agent name	Target	Mode of Action	Reference	Pipeline
Spliceostatin	SF3b complex	SF3b inhibitor, inhibits in-vitro splicing and promotes nuclear retention of pre-mRNA, influences selected splicing events	PMID: 17643111	Laboratory
Pladienolide	SF3b complex	SF3b inhibitor, regulates branch site recognition and selection during pre-mRNA splicing; influences selected splicing events	PMID: 17643112	Laboratory
E7107	SF3b complex	SF3b inhibitor, prevents binding of U2 snRNP to pre-mRNA; influences selected splicing events	PMID: 24258465	Phase I
H3B–8800	SF3b complex	SF3b inhibitor, modulates selected splicing by interfering with the interaction between the SF3b complex and branchpoint region	PMID: 34172893	Phase I
Spinraza ASO	SMN2	Promotes alternative splicing of SMN2 in turn produce SMN2 protein	PMID: 23027901	FDA Approved SMA
Eteplirsen ASO	Dystrophin	Triggers removal of Exon 51 in mutated dystrophin protein to restore functional dystrophin	PMID: 27929755	FDA Approved DMD
Selinexor	XPO1	Binds XPO1, causes nuclear accumulation of some factors and reduces eIF4E-XPO1 dependent RNA export	PMID: 34132444	FDA Approved refractory Multiple Myeloma & refractory Diffuse Large B-Cell Lymphoma
Eltanexor	XPO1	Binds XPO1 and inhibits eIF4E-XPO1 mediated nuclear export	PMID: 34817872	Phase I
Ribavirin	eIF4E	eIF4E cap competitor, impairs multiple eIF4E functions	PMID: 30478448	Phase I/phase II
7-BnGMP	eIF4E	eIF4E inhibitor, competes for binding to capped mRNA specifically	PMID: 19351181	Laboratory
4EGI1	eIF4E	eIF4E inhibitor, inhibits formation of 4E-4G complex	PMID: 26170285	Laboratory
CGP57380	MNK	MNK inhibitor, blocks eIF4E phosphorylation	PMID: 27289018	Laboratory
eFT508	MNK	MNK inhibitor, blocks phosphorylation of eIF4E	PMID: 39576211	Phase I

In the future, abnormal splicing in cancer could be targeted with RNA interference and antisense oligonucleotides (ASOs) (Table 1). The first clinical examples of therapeutic targeting of splicing came from the rare genetic diseases spinal muscular atrophy (SMA) and Duchenne muscular dystrophy (DMD) [117,118]. SMA is caused by insufficient SMN protein production arising from mutations or deletions in the *SMN1* gene. To compensate for this, SMN2 protein levels can be increased by obviating the skipping of exon 7 [119]. FDA-approved Spinraza promotes the inclusion of exon 7 by binding ISS (Intronic Splicing Silencer) motif in intron 7 of the SMN2 gene and prevents the inhibitory function of this motif on exon7 inclusion. By increasing exon 7 inclusion, Spinraza leads to production of functional SMN2 protein levels [117]. Similar strategies have been used for FDA-approved Eteplirsen (Table 1), an ASO, which binds the ESE motif in exon 51 of mutated DMD protein and influences it to be skipped by effectively hiding from spliceosome [118]. As a result, restoration of the translational reading frame of DMD leads to the production of shortened but functional dystrophin protein [118]. While these ASOs are not treating cancers, they provide proof-of-principle examples for developing similar strategies to target RNA splicing, particularly with new nanoparticle delivery systems now available [120]. However, ASO could have some challenges in cancer treatment, as alternative splicing occurs in hundreds of genes with concurrent disease mutations and possible off-target delivery of the ASO. Off-target effects result from complementary binding between ASO with non-targeted mRNA with a similar sequence of targeted mRNA [121].

As discussed above, eIF4E plays multiple roles in splicing, export, and translation [4]. Moreover, eIF4E drives oncogenic effects, and its dysregulation is associated with poor outcomes [18,19,25,26,39,40,122]. Thus, effective inhibitors would ideally inhibit its multiple functions simultaneously. Ribavirin, an old antiviral drug and the first pharmacological eIF4E inhibitor studied in the clinic, competes for m^7^G-cap of capped mRNAs as confirmed by NMR, mass spectrometry, and other biochemical techniques [46,123–137] (Table 1). Ribavirin inhibits eIF4E’s roles in splicing, mRNA export, and translation, while its functions in CPA and capping are yet to be tested [14,40,46,127–137]. The first clinical trial to target the therapeutic potential of targeting eIF4E used ribavirin monotherapy in relapsed/refractory AML, where 6/15 patients achieved objective clinical responses, including complete and partial remissions and blast responses (defined as a reduction in leukaemia cells by 50%) [18]. In patients, ribavirin reduced the nuclear localization of eIF4E, by losing its interactions with Importin 8 which in turn impaired its mRNA export function, which strongly correlated with clinical responses [18,19,41,138]. Ribavirin combined with low-dose cytarabine yielded similar results to monotherapy but with potentially longer responses [19]. Resistance to ribavirin was linked to its impaired cellular uptake or its glucuronidation [19,125,139–141]. Combining ribavirin with Vismodegib, which impaired glucuronidation [125,139], showed promise in overcoming glucuronidation-mediated ribavirin resistance in heavily pre-treated patients and cell lines [41,125,139]. In HPV-related stage IV oral cancers, ribavirin combined with afatinib and low-dose chemotherapy achieved partial remissions (6/10 patients) with reduced toxicity compared to standard chemotherapy [24]. Ribavirin monotherapy in oral cancers reduced phospho-eIF4E levels in 4/6 patients but provided limited therapeutic benefit, with the best outcomes being stable disease lasting up to ~7 months [142] suggesting eIF4E inhibition may be best in combination. Reduced phosphorylated eIF4E by ribavirin treatment impaired mRNA export function and translation of specific mRNAs involved in cell proliferation and survival [122,129]. ASOs targeting eIF4E showed efficacy in prostate cancer mouse models driven by elevated eIF4E expression; the second-generation ASO was used, retaining the phosphorothioate backbone core to initiate RNase H-mediated degradation of target mRNA and which is flanked with five 2-Methoxy methyl modified (MOE-modified) to improve strength, nuclease resistance, and tissue half-life [30]. However, this treatment failed to achieve objective clinical benefits in solid tumour patients, likely due to insufficient reduction of eIF4E protein levels in the patients [143]. However, this may be overcome with new nanoparticle delivery systems to better deliver the eIF4E ASOs to the target malignant cells. Phosphorylation of eIF4E contributes to its mRNA export and oncogenic activity [144]. MNK inhibitors, which target eIF4E phosphorylation, are currently under clinical investigation, with results pending (ClinicalTrials.gov NCT02040558). Other eIF4E inhibitors are in development or serve as laboratory tools, such as 4GI1 [145] (Table 1). Overall, while ribavirin and other eIF4E-targeting strategies show promise, optimizing combination therapies and addressing resistance mechanisms remain critical for achieving durable responses in patients.

Given the crosstalk and multiple activities of eIF4E, SF3B1, and U2AF1, it would be interesting to examine if a combination therapy of ribavirin, splicing inhibitors, and/or export inhibitors would be more efficient. Indeed, new laboratory studies explored the potential of targeting the nuclear export receptor XPO1 as a therapy for SF3B1-mutant MDS and AML [146]. Importantly, XPO1 is the nuclear export receptor used in eIF4E-dependent RNA export, and the activity of Selinexor, an XPO1 inhibitor, is in part attributed to its role as an inhibitor of eIF4E-dependent export of oncogenic RNAs [147,148] (Table 1). While SF3B1 mutations in MDS are generally linked to a good prognosis [103] elevated XPO1 expression in these patients is associated with worse survival [146,149]. XPO1 inhibitors, Selinexor and Eltanexor showed increased efficacy in SF3B1-mutant (SF3B1 K700E and K666N) MDS patients in phase I/II clinical trial [146]. In the future, it would be interesting to employ ribavirin as a therapy that targets all eIF4E’s biochemical functions in SF3B1-mutant patients to dissect if targeting eIF4E activities beyond RNA export will synergize with disrupted SF3B1 function to reduce cell proliferation.

## Conclusions, open questions and future directions:

**Figure 6.**
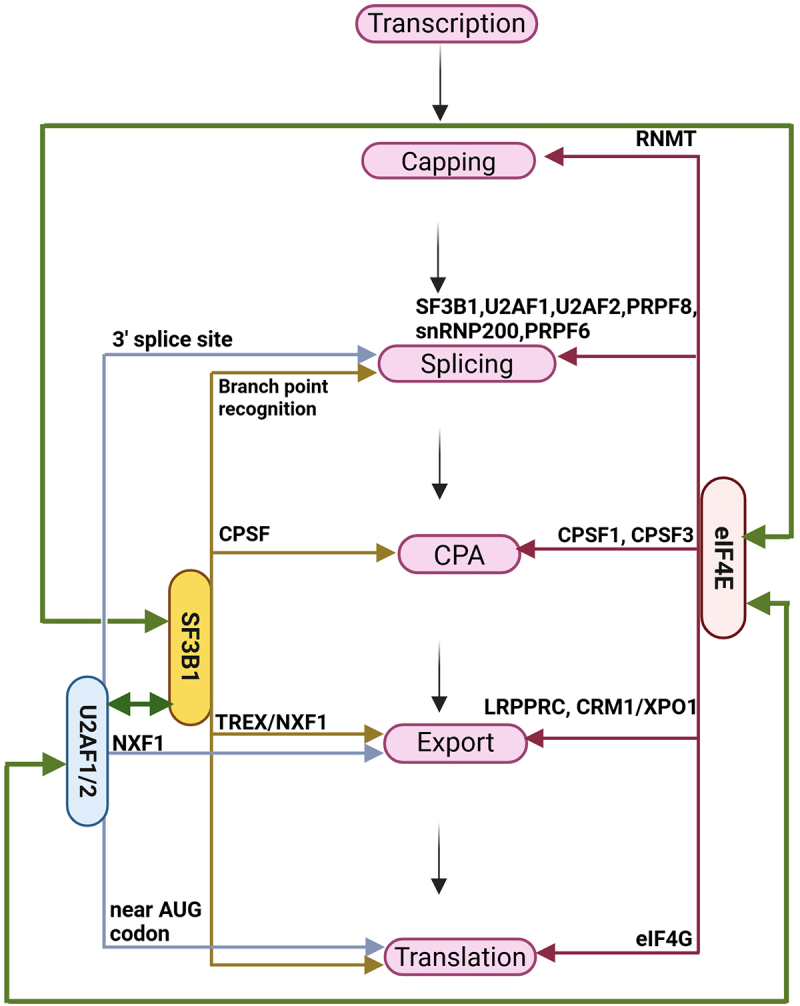
Schematic diagram highlighting multiple roles and the intersectionality of eIF4E, SF3B1, and U2AF1/2 in various mRNA processing steps by interacting with different processing factors [4,34,40,43,44,63,72–77,107–110]. Green lines indicate the functional interplay and interactions of these three proteins. Figure was generated in Biorender.

Here, we describe mechanisms for the simultaneous, broad-spectrum changes to multiple events of mRNA processing in AML and associated malignancies. We focused on eIF4E, SF3B1, and U2AF1 as example factors with multipurpose RNA processing capabilities (Figure 6). This likely relies on their capacity to directly select specific RNAs in different contexts and attract co-factor RBPs. Understanding the underpinnings of this multi-tasking by factors discussed here is relevant to predicting diversified proteomes and identifying potential therapeutic benefits and toxicities.

While these three example factors use conserved structural domains to interact with mRNA, the selection of targeted mRNAs differs in an RNA-processing event-specific manner. For example, U2AF1 binds defined sequence elements in the 3’ splice site (Figure 2), but during translation, U2AF1 recognizes what appear to be different sequence elements near the start codon [76] (Figure 5). Similarly, eIF4E binds the m^7^G cap found on mRNAs but selects different targets for splicing, export, and translation based on their USER code content and the presence of co-factors that recruit these into eIF4E-dependent complexes such as LRPPRC, the 4ESE RNA, and XPO1[45,63]. Whether SF3B1 directly binds to mRNAs during export when it is associated with TREX and NXF1 [107] is not yet clear, but it would be an important area to pursue finding the specific USER codes for other multi-tasking factors. It would be of great interest to know if the impacted RNA binding sites are conserved or differ for SF3B1 between splicing, export, and histone processing [107]. Moreover, eIF4E, SF3B1 and U2AF1 may target RNAs with specific USER codes directly or using co-factors and thereby elicit impacts on these mRNAs under selected processing events. Defining the intersection of co-regulated mRNAs between the three example proteins would provide a model for more complex networks and understanding whether physical interactions among eIF4E-SF3B1-U2AF1 are restricted to splicing or other events. Basic questions arise, such as whether these three factors bind to the same mRNAs at different positions when they act together; also, do they bind sequentially or simultaneously? Developing a deeper mechanistic understanding is important to predict the outcomes regarding the functional proteome and its ultimate impacts on normal and malignant cell physiology.

While eIF4E, SF3B1, and U2AF1 physically interact with each other in the nucleus, it appears likely they manifest a combination of distinct and cooperative functions, and these need to be better dissected (Figure 6). For example, eIF4E and SF3B1 are both involved in mRNA export but in different pathways. eIF4E associates with XPO1 and selects mRNAs with the 4ESE element; genetic reduction of eIF4E does not influence NXF1-mediated export and NXF1 knockdown does not impede eIF4E-dependent export [44,45,63]. By contrast, SF3B1 binds to NXF1, and the genetic reduction of SF3B1 impairs the NXF1-dependent mRNA export pathway for some RNAs [107]. However, it is possible that SF3B1 also acts in XPO1-mediated export; it would be interesting to ascertain in the future if this relies on eIF4E. Interestingly, pharmacological inhibition of XPO1 is more impactful in cells with SF3B1 mutation, in the limited studies to date, suggesting a genetic interaction. Moreover, as described above, eIF4E can influence SF3B1 and U2AF1 activity by driving their protein production via enhanced RNA export. eIF4E can increase the levels of multipurpose factors, providing another mechanism by which to integrate and coordinate multipurpose RBPs to programme the mRNA processing-export-translation axis. While these highlighted factors all influence splicing, it is interesting that they have overlapping but differing profiles regarding the targeted mRNAs.

All in all, the mRNA processing, export and translation pipeline is coupled through multi-tasking factors to mould the final form of mRNA and the ultimate protein. Understanding the complexity of how dysregulation of these factors impacts each other, and RNA processing is important for developing effective therapeutics for cancers such as AML, which are characterized by disrupted RNA processing, export, and translation. A mechanistic understanding of how dysregulation of these factors diversifies the malignant proteome will be imperative for developing future therapies.

## Data Availability

Data sharing is not applicable to this article as no new data were created or analysed.
